# COVID-19 and male fertility: short- and long-term impacts of asymptomatic vs. symptomatic infection on male reproductive potential

**DOI:** 10.3389/frph.2024.1403143

**Published:** 2024-05-23

**Authors:** Ahmad Majzoub, Kareim Khalafalla, Mohamed Arafa, Walid El Ansari, Arun Nair, Ahmad Al Bishawi, Mulham Saleh, Mohamed Khair Ella, Haitham ElBardisi, Muhammad Abu Khattab, Khalid AlRumaihi

**Affiliations:** ^1^ Department of Urology, Hamad Medical Corporation, Doha, Qatar; ^2^ Department of Clinical Urology, Weill Cornell Medicine—Qatar, Doha, Qatar; ^3^ Department of Andrology, Cairo University, Cairo, Egypt; ^4^ Department of Surgery, Hamad Medical Corporation, Doha, Qatar; ^5^ College of Medicine, Qatar University, Doha, Qatar; ^6^ Department of Population Health, Weill Cornell Medicine—Qatar, Doha, Qatar; ^7^ Department of Medicine, Museaid Hospital, Hamad Medical Corporation, Doha, Qatar; ^8^ Department of Infectious Diseases, Communicable Disease Center, Hamad Medical Corporation, Doha, Qatar; ^9^ Department of Medicine, Um Garn Quarantine Facility, Hamad Medical Corporation, Doha, Qatar

**Keywords:** COVID 19, reproductive hormones, semen quality, male fertility, SARS-CoV-2

## Abstract

**Background:**

Studies exploring the effect of COVID-19 on male reproductive system suggest a detrimental association, however with conflicting results. The aim of this study was to assess the association between COVID-19 infection and male reproductive potential including hormone profiles and semen parameters.

**Methods:**

This prospective cohort study included 48 patients with confirmed COVID-19 infection. Patients were subdivided into an asymptomatic group (*n* = 30) and a group with COVID-19 symptoms (*n* = 18). Serum hormone levels including testosterone, LH, FSH and estradiol were collected during active infection (baseline, time 0), and at 3 and 6 months following COVID-19 infection. Semen samples (basic semen analysis and oxidation reduction potential) were examined at 3 and 6 months following infection. Student and paired-t tests were used to compare continuous variables between the study groups and across the studied time intervals, respectively. Multivariate binary logistic regression analysis was performed to explore predictors for COVID-19 symptoms during active infection.

**Results:**

Patients with COVID-19 symptoms were significantly older (*p* = 0.02) and had significantly lower serum testosterone levels (*p* = 0.01) and significantly higher LH: testosterone ratio (*p* = 0.01) than asymptomatic patients. Multivariate analysis revealed older age (OR =  1.18, *p* = 0.03) and lower serum testosterone level (OR = 0.8, *p* = 0.03) as independent predictors of symptomatic COVID-19 infection. Significant increase in testosterone (*p* < 0.001 for both) and decrease in LH (*p* = 0.02, *p* = 0.007) and LH: testosterone (*p* = 0.02, *p* = 0.005) levels were observed at 3 and 6 months in patients with COVID-19 symptoms. Asymptomatic patients demonstrated significant increase in testosterone (*p* = 0.02) and decrease in LH: testosterone (*p* = 0.04) levels only at 3 months following COVID-19 infection. No significant differences were observed between the two study groups with regards to the semen analysis results obtained at 3 or 6 months following COVID-19 infection.

**Conclusion:**

Significantly lower testosterone values are associated with worse disease severity among men with COVID-19 infection. This association appears to be temporary as a significant increase in testosterone levels are witnessed as early as 3 months following recovery. No significant detrimental effect for COVID-19 infection on testicular sperm production is found in this patient population.

## Introduction

Since December 2019, the COVID-19 virus pandemic impacted people's lives and wellbeing, infecting more than 700 million people in 230 countries and territories, with mutations that led to variable disease severity. Typical symptoms include fever, sore throat and myalgia, but severe symptoms such as pneumonia and respiratory distress can inflict major morbidity and mortality. As of April, 2024, more than 7 million deaths have been directly associated with the pandemic (1).

Coronaviruses comprise a large family of viruses that are classified into Alpha, Beta, Gamma and Delta generations. Beta group constitutes the well-known SARS-CoV and MERS-CoV viruses, as well as the latest 2019 SARS-CoV-2 (2). Both SARS-CoV viruses utilize the angiotensin converting enzyme 2 receptor (ACE2) for tissue entry and depend on cellular transmembrane protease serine 2 for cleavage and activation of the spike protein, a necessary step for membrane fusion (3).

The scientific community was driven by the rapid progression of COVID-19 and numerous publications were released unraveling various aspects of this disease including its infectivity, clinical severity, and impact on various organ systems (4). With respect to the reproductive system, the expression of ACE2 receptors in testicular tissues including seminiferous tubules, germ cells, Sertoli and Leydig cells has been suggested as a plausible explanation for the virus's potentially detrimental impact on male fertility (5). Duarte-Neto et al. (6) analyzed postmortem testicular samples with reverse-transcription polymerase chain reaction (RT-PCR) for RNA detection and with light and electron microscopy (EM) and identified viral particles in the cytoplasm of various cellular contents of the testis. The authors also revealed that the impairment in testicular function might occur secondary to SARS-CoV-2 local infection and result from a combination of orchitis, vascular changes, basal membrane thickening, Leydig and Sertoli cell scarcity, and reduced spermatogenesis (6). The same group provided another explanation for spermatogenic dysfunction which might arise from subclinical epididymitis which was reported in 42.3% of hospitalized patients with COVID-19 infection (7). The authors identified radiographic patterns including disseminated micro-abscesses and inhomogeneous echogenicity with reactionary hydrocele existing in COVID-19 patients in the absence of testicular complaints, proposing that scrotal ultrasound for patients with mild-moderate infection (7). Others have attributed testicular damage to the high grade febrile illness associated with infection, and the inflammatory autoimmune testicular response which appears to mimic mumps viral orchitis (8). Finally, disruption of the hypothalamic-pituitary-gonadal axis was another proposed pathophysiologic mechanism linking COVID-19 infection to testicular endocrine imbalance (9, 10).

The literature reveals knowledge gaps. Few studies have observed an indirect association between serum testosterone and COVID-19 disease severity (11–13), however, a cause-effect relationship has not been fully established. In addition, results on the existence of the virus in testicular tissues or the impact of infection on semen parameters have been conflicting (14–17). To the best of our knowledge, no previous study has examined the association between COVID-19 infection and testicular function among patients from our geographic location.

The aim of the current study was to assess the associations between COVID-19 and several male reproductive parameters including a range of serum hormone levels (i.e., testosterone, luteinizing hormone, follicular stimulating hormone, and estradiol), as well as a number of semen features (macroscopic parameters, sperm concentration, total sperm count, total and progressive motility, sperm morphology and seminal oxidative stress). These associations were explored at different time points (0, 3 and 6 months), and among patients with different severities of COVID-19 infection (asymptomatic vs. COVID-19 symptoms). We also explored the clinical factors associated with the presence of symptomatic COVID-19 infection.

## Methods

### Study design and participants

This prospective cohort study was conducted at Hamad Medical Corporation hospitals and Quarantine facilities in Qatar between May 2020 and May 2021. Ethical Approval from the Institutional Review Board was obtained (IRB MRC-05-052) for the protocols and procedures of the study and all participants signed a written informed consent before their participation.

Inclusion criteria were unvaccinated patients who tested positive for COVID-19 infection and who had documented evidence of fertility (live birth) in the last 2 years before their infection. Patients with comorbid conditions including diabetes mellitus, hypertension, dyslipidemia, cardiovascular disease, respiratory disease, pre-existing infertility, or those with abnormal semen parameters who had received fertility treatment/s or testosterone replacement prior to the virus infection were excluded from the study. Unconscious or severely ill intubated patients during the infection were also excluded. No formal sample size calculation was conducted. We planned to involve all patients meeting the inclusion and exclusion criteria who agreed to participate in this study during the abovementioned timeframe.

### Procedures and outcomes

Patients were followed up from the time of COVID-19 diagnosis (baseline, time 0), and then after 3 and 6 months respectively. Real time PCR cycle threshold (CT) was used to determine the viral load and disease activity. Low CT indicated higher concentration of viral genetic material and activity (18). Serum hormone levels including testosterone (reference values 10.4–35 nmol/L), luteinizing hormone (LH, reference values 1–9 IU/L), follicular stimulating hormone (FSH, reference values 1–19 IU/L) and estradiol (reference values 73–275 pmol/L) were collected at time 0, and again at 3 and 6 months following COVID-19 infection. Samples were obtained from each participant between 7:00 and 9:00 am and the analysis was undertaken in the endocrine laboratory of our facility using the immunoassay chemiluminescence method, Architect i1000SR® (Abbott systems, Illinois, USA).

Semen samples were collected at 3 and 6 months following documented COVID-19 infection. Baseline (time 0) semen samples could not be obtained as patients were either quarantined or symptomatic. Each semen sample was assessed for macroscopic parameters such as color, pH, ejaculate volume, age of the sample, and viscosity after complete liquefaction. Based on the WHO fifth edition guidelines, an aliquot of the sample was examined for sperm concentration, total sperm count, total and progressive motility as well as sperm morphology (19). The samples were analyzed manually using a hemocytometer for sperm concentration assessment. Sperm motility was evaluated and categorized as progressive or non-progressive. Morphological evaluation was performed using the Diff-Quik staining protocol; 4% normal morphology was used as a cut-off based on Kruger's strict criteria (15). Seminal oxidative stress (OS) was assessed by measuring the static oxidation-reduction potential (sORP) of neat, liquefied semen samples using the MiOXSYS System (Caerus Biotech, Geneva). Details of the testing process have been published elsewhere (20).

Patients' age, number of offspring and their ages, serum hormone and semen parameter results were recorded. Patients were categorized into two study groups according to the NIH COVID-19 treatment guidelines (21). The first group comprised patients who tested positive for SARS-CoV-2 infection but had no symptoms consistent with COVID-19 (asymptomatic group). The second group constituted those who demonstrated any of the various signs and symptoms of COVID-19 e.g., fever, cough, sore throat, malaise, headache, muscle pain, nausea, vomiting, diarrhea, and loss of taste/smell (symptomatic group). The primary outcome measure was to evaluate the short- and long-term impact of COVID-19 infection on the reproductive hormone profiles and semen parameters of healthy fertile men.

### Statistical analysis

Statistical analyses were performed using IBM Statistical Package for the Social Sciences (SPSS, version 25). Continuous variables were expressed by mean and standard deviation. Categorical variables were expressed by frequencies and percentage. Normal distribution of variables was tested using histogram and Shapiro-Wilk test. Pearson correlation assessed the relationship between various continuous variables. Student-t test compared the clinical and laboratory variables between the study groups. Paired-*t*-test assessed changes in laboratory variables at different time points. Multivariate binary logistic regression analysis using the forward method explored the predictors for COVID-19 symptoms during the active infection. *p*-values <0.05 were considered statistically significant with an acceptable margin of error of 5%.

## Results

A total of 48 patients met the inclusion/exclusion criteria and their data were used in the current analysis. The mean age was 35.1 ± 5.6 years. All patients were fertile and had a minimum of 1 and a maximum of 5 offsprings and the youngest child mean age was 13.1 ± 8.4 months. Thirty patients (62.5%) were asymptomatic, while the remaining 18 (37.5%) patients had COVID-19 symptoms. The mean CT value was 23.4 ± 5.2.

For the total sample, the means of the baseline (Time 0) hormone profile were: Estradiol 90.9 ± 38.1 pmol/L, FSH 4.1 ± 2.1 IU/L, LH 6.5 ± 3.1 IU/L, prolactin 294 ± 195.7, and testosterone 9.9 ± 4.7 nmol/L. Twenty seven patients (55.3%) had low testosterone levels during infection, and COVID-19 symptoms were reported by 15 (55.6%) of them, while 12 patients (45.4%) were asymptomatic (*p* = 0.015). The follow-up hormone assays revealed normalization of serum testosterone levels in 11 out of the 15 (73.3%) hypogonadal patients who had COVID-19 symptoms.

### Asymptomatic vs. symptomatic patients: demographic and reproductive hormones profiles

Age, CT value, and baseline reproductive hormone profile (time 0) were compared between asymptomatic vs. symptomatic patients (Table 1). Symptomatic patients were significantly older, had significantly lower serum testosterone levels and significantly higher LH: testosterone ratio than asymptomatic patients. No significant differences were noted in the CT score between the two groups.

**Table 1:** Asymptomatic vs. symptomatic patients: demographic data and hormone profiles.

	Asymptomatic (*n* = 30)	Symptomatic (*n* = 18)	*p*-value
Age (years)	33.6 ± 5.8	37.6 ± 4.3	0.02
CT value (cycles)	22.8 ± 5.3	24.4 ± 5.1	0.28
Estradiol (pmol/L)	96.3 ± 40.2	82.2 ± 33.7	0.22
FSH (IU/L)	4.2 ± 2.4	3.8 ± 1.5	0.49
LH (IU/L)	6.5 ± 3.5	6.4 ± 2.5	0.98
Testosterone (nmol/L)	11.4 ± 4.8	7.5 ± 3.5	0.01
LH: testosterone (IU/L: nmol/L)	0.7 ± 0.5	1.1 ± 0.7	0.01

Student *t*-test, FSH, follicular stimulating hormone; LH, luteinizing hormone; CT, cycle threshold.

### Asymptomatic vs. symptomatic patients: changes in reproductive hormones across 3 time points

**Figure 1:**
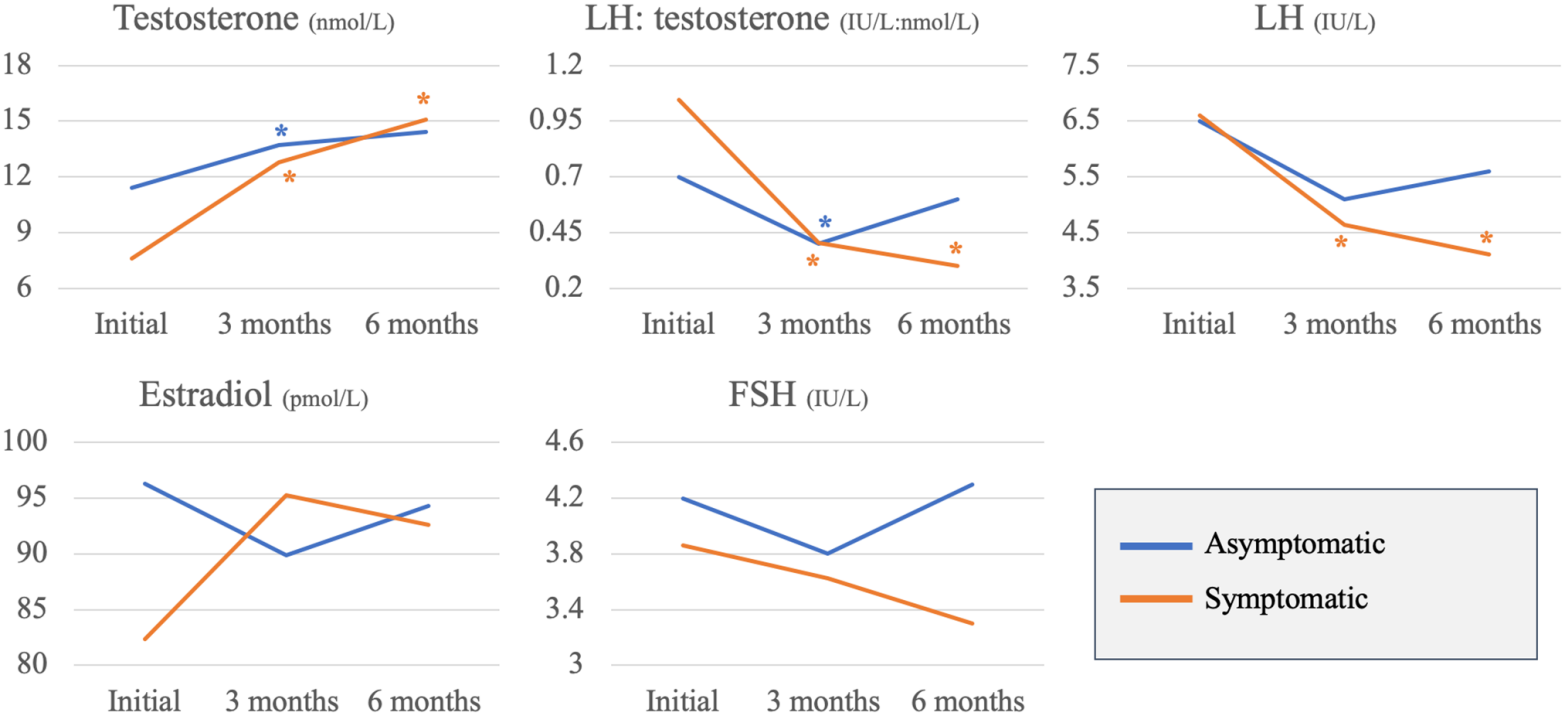
Asymptomatic vs. symptomatic patients: changes in reproductive hormones across 3 time points (*p-value <0.05).

Changes in the hormone profile were assessed 3 and 6 months following COVID-19 infection and compared between the two study groups. In comparison to the baseline hormone results, symptomatic patients had a significant increase in serum testosterone (7.7 ± 3.5_initial_ nmol/L; 12.9 ± 4.6_3months_ nmol/L, *p* < 0.001; 15.1 ± 5.3_6months_ nmol/L, *p* < 0.001), significant decrease in serum LH levels (6.6 ± 2.6_initial_ IU/L; 4.6 ± 1.4_3months_ IU/L, *p* = 0.02; 4.1 ± 1.1_6months_ IU/L, *p* = 0.007) and significant decrease in LH: testosterone ratio at 3 and 6 months following COVID-19 infection (1.1 ± 0.7_initial_ IU/nmol; 0.4 ± 0.2_3months_ IU/nmol, *p* = 0.02; 0.3 ± 0.2_6months_ IU/nmol, *p* = 0.005). On the other hand, asymptomatic patients had significant increase in serum testosterone (11.4 ± 4.8_initial_ nmol/L; 13.7 ± 4.7_3months_ nmol/L, *p* = 0.02) and significant decrease in LH: testosterone ratio (initial: 0.7 ± 0.5_initial_ IU/nmol; 0.4 ± 0.1_3months_ IU/nmol, *p* = 0.04) only at 3 months following the infection (Figure 1).

### Relationships between viral load/disease activity and age and reproductive hormonal profile

**Figure 2:**
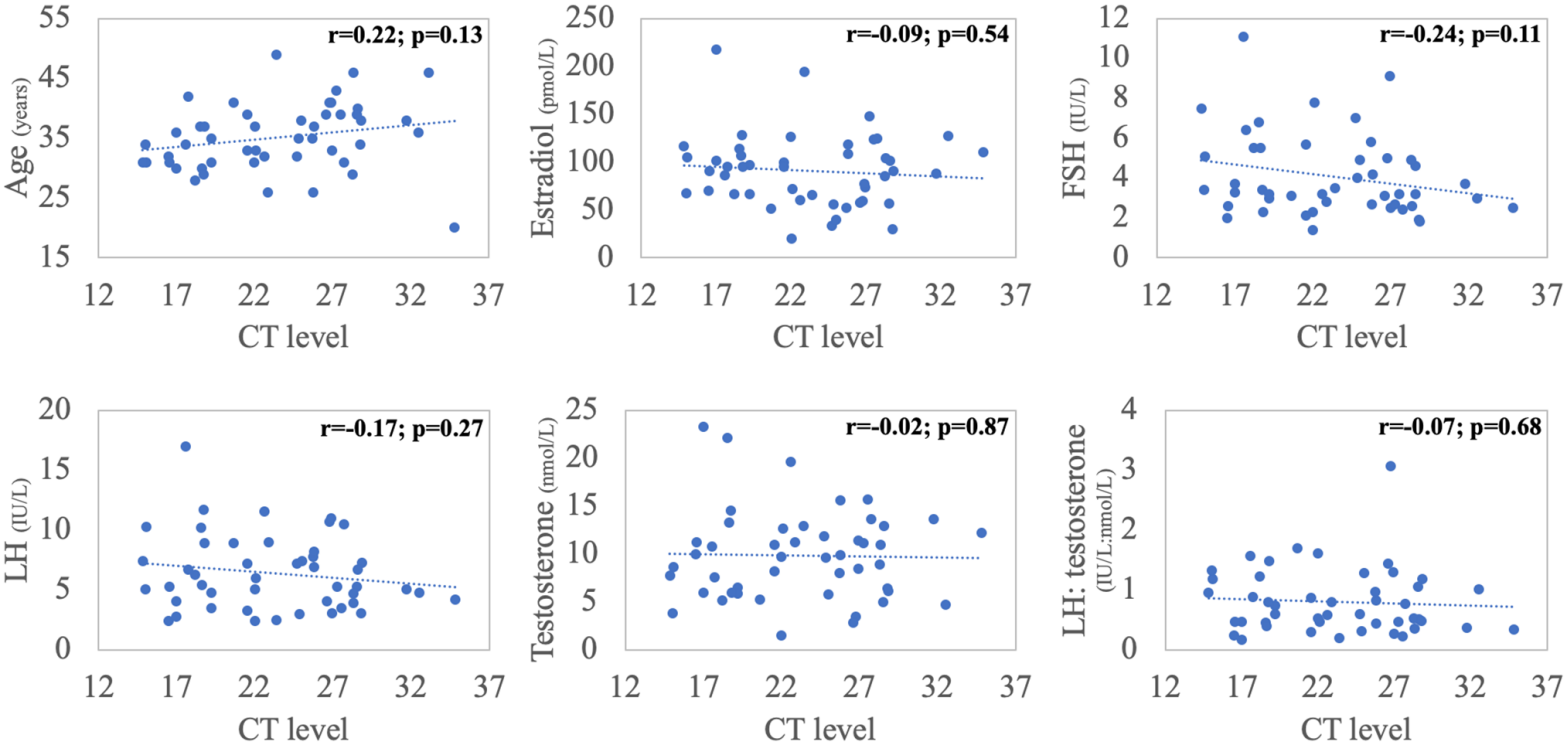
Correlations between cycle threshold (CT) value, patient's age and baseline (time 0) hormonal panel *(Pearson's correlations).*

Pearson's correlations were performed between the CT scores and patients' age and initial hormone profile. There were no significant relationships between CT level and age or any of the reproductive hormones (Figure 2). Multivariate binary logistic regression analysis revealed that older age [Adjusted odds ratio (aOR) = 1.18, 95% confidence interval (CI) = 1.02–1.36, *p* = 0.03] and lower serum testosterone level (aOR = 0.8, 95% CI = 0.65–0.97, *p* = 0.03) as independent predictors of symptomatic COVID-19 infection (Table 2).

**Table 2:** Regression analysis of the likelihood of development of COVID-19 symptoms.

	Adjusted odds ratio	95% confidence interval	*p*-value
Age (years)	1.18	1.02–1.36	*0* *.* *03*
CT value (cycles)	0.8	0.65–0.97	*0*.*03*
Estradiol (pmol/L)	—	—	0.26
FSH (IU/L)	—	—	0.55
LH (IU/L)	—	—	0.56
Testosterone (nmol/L)	—	—	0.48
LH: testosterone (IU/L: nmol/L)	—	—	0.60

Multivariate binary logistic regression using the forward stepwise model.

FSH, follicular stimulating hormone; LH, luteinizing hormone; — variables not included in the final model, italicized cells indicate statistical significance.

Semen analysis results obtained at 3 and 6 months following infection were compared between the two study groups. No significant differences were observed between the two study with regards to the semen analysis results obtained at 3 or 6 months following COVID-19 infection (Table 3). Further, the paired *t*-test analysis did not reveal any significant changes between samples obtained at 3 and 6 months following infection in each of the study groups.

**Table 3:** Changes in semen quality at 3 and 6 months following COVID-19 infection in asymptomatic vs. symptomatic patients.

Variable	Asymptomatic (*n* = 30)		Symptomatic (*n* = 18)	
	3 months	6 months	3 months	6 months
Semen volume (ml)	2.8 ± 1.4	2.3 ± 1.9	3.2 ± 1.8	2.7 ± 1.4
Concentration (10^6^/ml)	50.2 ± 30.9	52.4 ± 26.6	47.3 ± 10.4	42.3 ± 14.6
Total motility (%)	54.1 ± 6.7	55.1 ± 5.1	53.9 ± 6.9	54.5 ± 5.6
Progressive motility (%)	19.7 ± 9.5	19.7 ± 9.2	18.5 ± 7.2	17.8 ± 9.2
Normal morphology (%)	8.2 ± 3.9	8.2 ± 3.5	9.6 ± 4.9	8.4 ± 4.3
sORP (mVolt/10^6^ sperm)	1.8 ± 1.2	1.9 ± 1.9	1.5 ± 1.3	1.6 ± 1.1

Paired-*T*-test; sORP, static oxidation reduction potential.

## Discussion

This study assessed the association between COVID-19 infection and male reproductive hormones and semen parameters. Our main findings were that symptomatic COVID-19 patients had significantly lower serum testosterone and significantly higher LH: testosterone levels compared to asymptomatic patients. This result has been echoed by some studies documenting a significant relationship between different reproductive hormones and the severity of COVID-19 infection.

In terms of changes in reproductive hormones with COVID 19 infection, a case-control study (13) of 89 patients hospitalized for COVID-19 disease, 30 hospitalized patients with respiratory tract infection due to other causes, and 143 age matched healthy controls, revealed significantly lower testosterone and higher LH and prolactin levels in patients with COVID-19 infection compared with the other 2 groups. Furthermore, serum testosterone was found to be positively correlated with oxygen saturation and negatively correlated with patients' hospital duration only in the COVID-19 group (13). Another cross-sectional study by Okçelik et al. (22) of 44 outpatient COVID-19 positive subjects, revealed significantly lower testosterone levels among patients who developed pneumonia compared to those who did not. Rastrelli et al. (12) assessed the reproductive hormones of 31 intensive care unit patients diagnosed with pneumonia secondary to COVID-19 infection and identified significantly lower total and free testosterone and higher LH in severe/deceased patients compared to those who remained stable or who had clinical improvement. The authors speculated that lower baseline testosterone levels may predict mortality outcomes of COVID-19 patients in the intensive care unit (12).

Our multivariate regression analysis identified testosterone as a significant independent predictor of a worse COVID-19 disease course. This finding was also observed by Rastelli et al. (12) who conducted linear regressions between testosterone levels and various markers of disease severity and demonstrated that with each 1 mmol/L decrease in testosterone, the probability of having a worse outcome was increased by an OR of 1.42 (95% CI = 1.06; 1.89; *p* = .017) (12).

While the results of the abovementioned studies confirm a significant relationship between levels of reproductive hormones and COVID-19 disease severity, they do not imply causation. All previous reports were either observational or case control studies in which hormone assessment was only performed during active infection. The current study investigated changes in the hormone profile of recovered patients at 3 and 6 months following infection. Our results revealed significant improvements in testosterone, LH, and LH: testosterone ratio at 3 months following infection in all patients, and further improvements at 6 months, particularly in symptomatic patients. In addition, 73.3% of patients in the symptomatic group who had low testosterone levels during the active disease, showed normalization of their serum testosterone levels in the months following infection.

These results support a “vicious circle” hypothesis between reproductive hormones and COVID-19 infection. The SARS-CoV-2 virus adheres to and lowers the expression of ACE2 receptors on Leydig cells resulting in an increase in angiotensin II, thereby blunting LH induced testosterone production (23). This ultimately causes a decrease in serum testosterone levels and an increase in LH levels, a picture that has been observed by our study and others (12, 13). On the other hand, accumulating evidence supports the anti-inflammatory properties of testosterone which, during depletion, would result in an increase in pro-inflammatory cytokines and a reduction in lymphocyte differentiation (24, 25), both of which have been regarded as hallmarks of COVID-19 infection. In this sense, it is plausible to accept low testosterone, as a consequence of infection, could by itself contribute to the disease progression resulting in worse outcomes. This notion was supported by the work of Buonacquisto et al. (26) which aimed at identifying male prognostic factors for disease severity. The authors concluded that reduced androgen receptor activity (evidenced by excessive CAG polymorphisms ≥23 repeats) and low testosterone levels to be significant contributors of an excessive inflammatory response leading to multi-organ failure in patients with severe COVID-19 infection (26).

In terms of assessing the effect of COVID-19 infection on testicular spermatogenic function, studies using varied methodologies to assesses the effect of COVID-19 infection on semen quality, reported conflicting results (11, 14, 15, 17). Accurate consideration of the effect of COVID 19 infection on spermatogenesis and its impact on conception must compare semen parameters pre-/post-infection among men with normal fertility potential. The literature depicts several shortcomings that may have been imposed by the pandemic nature of the disease and the difficulty in obtaining semen samples from sick infected men.

To illustrate, few studies compared the results of post COVID-19 semen samples with those obtained before infection (11, 14, 15, 17, 27, 28) and for the few that did, most pre-infection samples were those of infertile men seeking treatment. Two studies (*n* = 29 and 3 patients) did not find significant differences in semen quality before and after infection (15, 11). Conversely, Pazir et al. (14) (*n* = 24) detected significant reductions in sperm total motility within 4 months following COVID-19 infection compared with pre-infection results, although the average post-infection total motility was within the normal range. Zhang et al. (27) explored changes in semen parameters among 85 patients who had COVID-19 infection. Only 34 patients had semen results before and 3 months after the infection while 13 patients had a subsequent semen analysis performed 3–6 months after recovery. The authors observed significant reduction in sperm concentration and total sperm count within 3 months following the infection, however, with a median that fell within the normal reference range. Moreover, a significant increase in sperm concentration and total sperm count was observed following recovery. Erbay et al. (17) compared patients with mild (*n* = 26) vs. moderate COVID-19 symptoms (*n* = 43) up to 190 days following recovery and revealed significant reductions in progressive and total motility in the mild COVID-19 group compared to pre-infection results. Furthermore, significant reductions in semen volume, sperm concentration, progressive and total motility were observed in the moderate COVID-19 group in comparison to pre-infection results. However, we observed that Erbay's pre-infection results for some parameters appeared to fall below the reference values while some post-infection results fall within the normal reference values (17).

Other research employed appropriate study populations by including men with no prior infertility, comparing semen during active infection vs. following recovery. Falahieh et al. (29) (*n* = 20) reported below normal values for progressive/total motility during active infection and revealed significant improvements to normal thresholds in both parameters up to 120 days following recovery. Similarly, others (16) reported significant improvement in sperm concentration in samples obtained following recovery in comparison to those obtained during the active infection in 22 men. We followed the same study design, where our patient population included men with proven fertility prior to COVID-19 infection as shown by natural pregnancy of their spouses within 24 months before infection. We assessed semen at 3 and 6 months post-infection but not during active infection because of quarantine regulations. Semen quality of our patients was within the normal reference ranges at both 3 and 6 months post-infection. In addition, no significant differences were observed between the two study groups or the two time points of analysis. Our results coincide with the findings of previous studies (16, 25, 28) indicating that even if a detrimental effect on sperm production occurs during the active disease, the impact is not persistent, and recovery could be expected within 3 months following the infection. With respect to OS measures, our study did not find any significant differences in the *sORP* levels between the semen samples obtained 3 and 6 months following infection. OS is a well-recognized intermediary state that occurs secondary to inflammation and cytokine release, and has a considerable impact on fertility as it accelerates lipid peroxidation, abortive apoptosis and sperm DNA fragmentation (30). Falahieh et al. (29) assessed seminal reactive oxygen species levels, lipid peroxidation and total antioxidant capacity during active infection (within 14 days) and 120 days following recovery. The authors did observed significantly higher OS measures during active infection in comparison to the samples obtained during the follow-up. Unfortunately, we were not able to examine OS measures during active infection and our initial semen results coincide with the follow-up data of Falahieh et al. (29) further advocating the lack of persistent effects for SARS-COV-2 infection on semen quality.

This study has limitations. A larger sample size would have been beneficial but was hindered by the infective nature of the disease, the national restrictions, and attitudes toward containing the pandemic. However, our sample size was comparable to others reported in the literature (11, 16, 22). Examining semen parameters during the initial infective phase of the disease would have been useful in documenting the baseline semen status. Although this limited understanding the short-term effects of the COVID-19 infection on spermatogenesis, however, it provided an opportunity to eliminate the effect of the febrile illness on semen parameters, and thus, examine any independent effect of COVID-19 virus on spermatogenesis. Additionally, it would have been interesting to explore the effect of COVID-19 infection on other aspects of male reproductive health including erectile and ejaculatory function. The study also has many strengths; it was prospective, the assessment of hormonal profile at three time points provides more compelling evidence towards causation, and the multivariate analysis explored the independent predictors of disease severity.

## Conclusion

There appears to be a reciprocal effect between COVID-19 infection and endocrine function of the testis that is more intense with increasing COVID-19 severity. Serum testosterone was significantly lower in patients with COVID-19 symptoms compared to asymptomatic patients and was also a significant independent predictor of worse disease severity among men with COVID-19 infection. For most of these patients, the hypogonadal state observed during active infection reversed back to an eugonadal state as early as 3 months following recovery.

## Data Availability

The raw data supporting the conclusions of this article will be made available by the authors, without undue reservation.
